# The Certified Mental Health Assistant: An Innovative Solution to a Growing Crisis

**DOI:** 10.1007/s40596-025-02244-1

**Published:** 2025-10-07

**Authors:** Randon S. Welton

**Affiliations:** Northeast Ohio Medical University, Rootstown, OH USA

Despite recent increases in psychiatry training slots, the number of psychiatrists in the USA will significantly decline over the next decade as older psychiatrists leave the workforce. Of the 40,000 psychiatrists working in 2023, 36% were 65 years of age or older [1]. With the declining workforce and increasing demands, the Health Resources and Services Administration predicts that by 2037, Ohio will have less than 40% of the necessary general psychiatrists [2]. The shortage already impacts community mental health centers. A statewide survey found that 98.6% of responding organizations had difficulty hiring new staff, and 88.3% had difficulty retaining current staff, especially clinical and medical staff [3]. These shortages likely influence patients’ satisfaction with access to care and perceived quality of care, both of which are lower in Ohio than national averages [4].

As state legislators and leaders in medical education considered solutions, most fell into the category of “do more of what we are currently doing.” Training more psychiatrists is long and expensive and places the funder at risk if graduates choose to leave the state. Training more psychiatric physician assistants or mental health nurse practitioners could be valuable, but limitations to their scope of practice in Ohio would lead many graduates to practice in states where they have more autonomy. These are praiseworthy but insufficient responses. State legislators and medical educators proposed a novel solution, the certified mental health assistant (CMHA) based on lessons learned in creating certified anesthesiologist assistants.

## Overview of the Certified Mental Health Assistant Program

The CMHA position is based on a 2-year master’s degree, which focuses exclusively on managing medications in individuals with mental illness and substance use disorders. The first year is largely classroom-based and will include in-depth, interactive discussions of the anatomy, physiology, pathology, and pharmacology involved in managing mental illness and substance use disorders. Training during this year will feature case-based and problem-based learning. Clinical skills and clinical reasoning will be emphasized, and there will be a thorough introduction to evidence-based medicine in the form of published clinical practice guidelines and treatment algorithms. CMHA students will be expected to apply these algorithms and guidelines to realistic clinical vignettes and scenarios. The second year will largely consist of supervised clinical experiences on inpatient mental health units, outpatient mental health clinics, and substance use disorder treatment centers. These clinical experiences will be augmented by psychiatrist-led discussions of more complex and subtle clinical vignettes. While the emphasis will be on medical management, the CMHA students will be instructed in motivational interviewing and supportive therapy. These will be taught in a seminar fashion, but will be practiced using clinical vignettes, role-playing, and standardized patients in the first year and provided under supervision on clinical rotations.

Safeguards were created to ensure the safety and quality of care provided by CMHAs. Patients must be evaluated and diagnosed by a physician before seeing a CMHA. After a thorough biopsychosocial evaluation, the physician will decide which patients are appropriate for someone with a CMHA’s level of training and experience. The physician must meet with the CMHA weekly to review cases and hear about patients exhibiting marked changes. Restrictions were placed on CMHA prescribing privileges. They can prescribe controlled substances only for physician-diagnosed disorders for which there is an FDA indication. The only opioid they can prescribe is buprenorphine for physician-diagnosed opioid use disorder. They are prohibited from prescribing psychedelic medications or from providing electroconvulsive therapy or transcranial magnetic stimulation. While CMHAs will receive training in geriatric and child/adolescent psychiatry, they will only see those patients if their supervising physician is comfortable supervising those patients. CMHAs’ prescribing practices will closely follow the clinical practice guidelines and treatment algorithms they were trained to use. The state medical board has been tasked with licensing CMHAs and overseeing the CMHAs’ requirement for continuing education in psychopharmacology.

During their training, CMHA students will receive over 700 hours of didactic and interactive training on the management of mental illness and substance use disorders and will have 8 months of supervised clinical care. This exceeds the training of most mental health nurse practitioners and psychiatric physician assistants. Three distinct aspects of CMHA training and practice further set them apart from other categories of mid-level prescribers.

## Creating a Rational Approach to Patient Assignment Using Certified Mental Health Assistants

Currently, individuals with mental illness connect with health care providers in an almost random manner. At least half of patients with depression or anxiety and up to a third with severe mental illness are managed by their primary care provider [5]. These primary care providers, which include physician assistants and nurse practitioners, often have limited formal training in mental health care. To complicate this issue further, HRSA estimates that by 2037, we will have less than 80% of the physicians required to meet the primary care needs of the nation [2] limiting their ability to help provide mental health care. Individuals seeking outpatient mental health specialty care are often frustrated in their efforts. Research in large urban areas has found that only 3–26% of calls by new patients result in the possibility of an appointment being made with a psychiatrist [6–8].

The presence of a CMHA creates a potentially new way of assigning patients. Every patient will be seen initially by a physician. Once the physician is comfortable with the diagnosis and basic treatment plan, they will then decide the appropriate level of care for that patient. If the problem is straightforward and the patient’s distress and impairment are mild, the patient can be managed by their primary care provider. If the patient’s acuity is high or the situation is complex, then they should be managed by a psychiatrist. There is, however, a large middle ground for providers who are not as highly trained as a psychiatrist but have received considerably more training in managing mental illness and substance use disorders than primary care providers. The CMHA will be the optimal level of care for patients with illnesses of mild to moderate severity and complexity. This triaging of patients establishes a rational approach to matching the severity and acuity of the patient’s problems with providers’ experience and training. For the psychiatrist, the CMHA will be a true physician extender. Studies looking at the Hamilton Depression Rating Scale have found that only 20–25% of patients with major depressive disorder have severe or extremely severe depression [9, 10]. The CMHA would likely be able to handle most of the other depressed patients. By taking patients with mild to moderate complexity and severity, CMHAs will allow psychiatrists to prioritize their time and focus on services that only a psychiatrist can provide: conducting comprehensive evaluations, teaching/supervising medical students and residents, consulting with other medical providers, and managing patients with more complex and complicated illnesses, allowing them to receive more frequent or extended appointments. In primary care, the CMHA will be a relative mental health expert, enhancing the primary care physician’s ability to provide mental health care. The CMHA’s presence will increase the efficiency of mental health care and allow all providers to practice at the top of their license.

## What and How Certified Mental Health Assistants Are Taught

Students matriculating into medical school do not know which specialty will ultimately interest them the most. They are therefore trained in all aspects of medicine, including numerous hours of training in mental health topics and several weeks on psychiatric clerkships. After having spent 4 years learning to be a general physician, future psychiatrists then receive specialized training. Much of what future psychiatrists labored to learn during their years in medical school will not be applicable to day-to-day psychiatric practice outside of consultation-liaison psychiatry. Most psychiatrists in outpatient clinics and psychiatric units manage only the patient’s mental illness and/or substance use disorders and leave all other medical issues to other providers. The training of CMHAs will exclusively concentrate on the knowledge and skills necessary to assist physicians in helping patients with mental illness and substance use disorders.

The deliberate focus of CMHA training on mental health issues makes CMHA training much more efficient. An example might involve what a mental health prescriber needs to know about the kidney. They would certainly need to know the laboratory studies that measure renal function and the impact of renal failure on mental functioning. They would need to have a thorough knowledge of medications that raise or lower lithium levels and the potential adverse impact lithium might have on renal functioning. That may not be the complete list, but it encompasses much of what mental health providers need to know about the kidney. CMHAs will not be trained to distinguish among the functions of the proximal tubule, distal tubule, or loop of Henle or to identify renal cells under a microscope, as those skills are not necessary in daily clinical practice. CMHAs will be trained to consider and assess for medical conditions that may exacerbate or mimic symptoms of mental illness, e.g., hypothyroidism, anemia, and cardiac arrhythmias. While the CMHA will not be trained to treat those conditions, they will, with the assistance of their supervising physician, identify them and refer them for more definitive care. Much of the training will be case-based. An example might be a patient who was doing well on their lithium but is now presenting with severe dizziness, nausea, and vomiting. The CMHA will be trained to obtain and interpret appropriate laboratory studies, diagnose lithium toxicity, and discuss which medications or circumstances might have caused lithium toxicity in a previously well-controlled patient. The CMHA will be trained to look for and identify the physical symptoms and common medical complications of substance use disorders, even though their management might end up with other providers. Similarly, the CMHA would be trained to recognize delirium, but once they have recognized delirium, the patient would be transferred to a psychiatrist whose broader and deeper training would be required.

Published entrustable professional activities (EPAs) will help guide the training of CMHAs. EPAs are discrete components of essential professional conduct that can be observed and assessed. There are published EPAs for graduates from medical school [11] and psychiatry residencies [12]. While the CMHA will not be able to perform all these activities, many can be incorporated into CMHA training as the foundation for their Educational Program Objectives. For example, the AAMC medical student EPA 6 is *Provide an Oral Presentation of a Clinical Encounter*. One of the key components is to “Provide an accurate, concise, well-organized oral presentation.” This EPA describes the trustworthy learner/provider as one who (1) filters, synthesizes, and prioritizes information into a concise and well-organized presentation; (2) integrates pertinent positives and negatives to support [their] hypothesis; and (3) provides sound arguments to support [their] plan [13]. These are goals for CMHA training. Incorporating select EPAs and their subordinate tasks into Educational Program Objectives for CMHA training will ensure that graduates have the necessary clinical skills to manage the patients they will be assigned and to approach patient care in ways familiar to their physician supervisors.

To emphasize the CMHA’s role on physician-led, multidisciplinary teams, psychiatrists will be an integral part of CMHA training. The Program Director and Medical Director for the program will both be psychiatrists with extensive experience in psychiatric care and education. Physicians will be involved in teaching clinical interviewing, motivational interviewing, and supportive therapy techniques. Psychiatrists will help teach clinical reasoning skills as well as how to create and narrow down a differential diagnosis. On the clinical rotations, CMHA students will be supervised by psychiatrists. Having physicians serve as integral components of the training will solidify the CMHAs’ role as physician extenders. In mental health settings, CMHAs will work closely with psychiatric supervisors and become a vital component of psychiatrist-led multidisciplinary mental health treatment teams, and in primary care settings, they will become trusted allies enhancing the knowledge and quality of care provided by the primary care team.

## How Certified Mental Health Assistants Are Assessed

Medical and psychiatric educators have been discussing competency-based approaches to training and assessment for decades. The last quarter of a century has seen a dramatic shift in accrediting bodies that had focused on structure and process measures but now seek to assess educational outcomes. This led to the development of core competencies and milestones [13, 14]. A competency-based approach to training requires frequent assessments and formative feedback to optimize learning, and robust summative assessments to determine promotion and advancement within the program [13]. The CMHA training program is being developed with this approach in mind. Knowledge-based examinations will still ensure that the basic material has been retained and understood, but the CMHA program will develop a system of overlapping assessments by specifically trained supervisors. Workplace-based assessments allow for a more authentic evaluation of how a trainee functions in real-life situations. Frequent assessments allow for greater opportunities to recognize where learners are struggling and their need for extra support/training [15].

Rather than being evaluated on their ability to recall memorized facts, the CMHA in non-clinical settings will be largely assessed on how they work through standardized vignettes and scenarios. In clinical settings, supervisors, patients, and medical team members will be asked how the CMHA performed discrete, specific tasks. The EPAs which are used to structure training can also be used to structure these assessments. Each EPA lists numerous, specific tasks. In class, the CMHA student will be presented with a series of clinical vignettes in which they will need to demonstrate these tasks to their instructors. On clinical rotations, supervisors will be asked to assess these tasks repeatedly until the CMHA student has demonstrated the ability to perform this function consistently and reliably. These assessments can be compiled into a portfolio of demonstrated competencies, which will be used to note where the individual CMHA needs improvement. If a CMHA student shows a specific deficit in their portfolio, the program leadership can work to determine the cause. If it is simply because the student has not had enough opportunities to demonstrate their ability, then additional interactions can be arranged. If they have had sufficient opportunities but have not completed them successfully, then the plan will include remediation of these skills followed by more clinical opportunities. The accumulated experiences in the portfolio can be used to inform decisions about advancement, promotion, and graduation.

Although workplace-based assessments helpfully shift the focus of trainee evaluation from what they know to what they do, concerns have been raised that will need to be addressed. Many trainees and supervisors see the requirement for direct observation of the trainee as intimidating and/or excessively time-consuming. Trainees and supervisors often do not understand the purpose of the assessments or fail to use them to provide formative feedback. Approaches to mitigating these concerns have been described and will be incorporated into the CMHA program. The importance of direct observation and formative feedback will need to be understood by trainees and supervisors. Schedules will need to be adjusted to allow time for direct observation and feedback. Trainees and supervisors will need to buy in to the concept that direct observations paired with actionable feedback are an effective way to improve skills and the quality of care provided [16–19].

Faculty development is a key component in the formation of the CMHA training program. Supervisors will need to fully understand the role and capabilities of the CMHA. New supervisors will need to be extensively instructed on what is expected of them. Supervisors will need to prioritize direct observation of the CMHA student at work and develop a shared understanding of what constitutes successful completion of each relevant task. Supervisors will need training and practice to ensure their feedback includes specific comments for improvement rather than general impressions of the CMHA’s work [13]. There will need to be an ongoing process to ensure consistency and standardization among supervisors. Supervisors will be brought together periodically to observe videotaped student interactions and discuss and reach consensus on what is adequate student performance.

## Conclusion

The development of the CMHA program has not been without its skeptics and critics. Often, concerns took the form of anecdotes about interactions with poorly trained mid-level providers. We hope to ameliorate those concerns by the innovative approaches to training, assessment, and practice that are described above. The extensive reliance on published clinical practice guidelines and treatment algorithms will help ensure that CMHA practice aligns with the best practices in the literature. The integral role psychiatrists have in training and assessing CMHA students also differentiates it from the training of other mid-level providers. This exposure to psychiatric practice during the 2 years of training will allow the CMHAs to become a cornerstone of physician-led multi-disciplinary teams.

Continuing, system-wide changes will be required to fulfill the potential of the CMHA program. Once the CMHA program was signed into law, Northeast Ohio Medical University (NEOMED) began working with the State Medical Board and Psychiatric Physicians Association to develop a finalized curriculum. Over time, NEOMED will be seeking feedback from supervisors and employers about the strengths and weaknesses of the CMHA training program. CMHA graduates will be assessed to ensure that their practices mirror the training they received and that they are continuing to follow the latest published guidelines. The CMHA program will provide invaluable assistance in addressing a public health emergency, the lack of trained psychiatrists, while we advocate for innovative ways of making medical education more affordable and for increasing the number of psychiatry residency training positions. Hopefully, leaders in academic psychiatry will embrace this opportunity to thoughtfully craft a new model for mental health care. Psychiatrists should play a key part in guaranteeing that patients receive comprehensive care, including psychotherapy and other psychosocial interventions, as well as evidence-based psychopharmacology. Universities and health care systems involved in this effort will need to ensure that academically oriented psychiatrists have the capacity to train and supervise CMHAs, as well as increased numbers of psychiatry residents. Cooperation and partnerships are necessary for us to address our burgeoning mental health crisis.

## Data Availability

The program described is being developed and, as of yet, has no data to be published. All of the other data mentioned in this commentary are from the referenced sources.
